# **High and strikingly early failure-rate following gram-negative periprosthetic joint infection – a retrospective cohort study on 72 cases**

**DOI:** 10.1007/s00402-026-06188-5

**Published:** 2026-02-02

**Authors:** Benjamin Schlossmacher, Bibiana Mathes, Vincent Lallinger, Dirk Mueller, Ruediger von Eisenhart-Rothe, Igor Lazic

**Affiliations:** Department of Orthopaedics and Sports Orthopaedics, Technical University of Munich, Munich, Germany

**Keywords:** Revision arthroplasty, Periprosthetic joint infection, Gram-negative

## Abstract

**Introduction:**

Periprosthetic joint infections (PJI) represent a major complication of total joint arthroplasty. While most infections are caused by staphylococci species, a notable proportion involves gram-negative bacteria. Due to the smaller numbers, outcome reports in literature are scarce and heterogenous success rates have been reported. This study aimed to (1) evaluate the overall treatment success of gram-negative PJI and (2) identify the most suitable surgical treatment strategy in eradicating gram-negative PJI.

**Materials and methods:**

Seventy-two cases of gram-negative PJI treated between 2010 and 2022 were analyzed in this retrospective cohort study. The median follow-up (IQR) was 18.0 (46.0) and at least 12 months. Outcomes were assessed based on the 2013 Delphi consensus on PJI outcome. PJI was defined according to the EBJIS-criteria.

**Results:**

The overall infection-free and revision-free survival rate was 43.1% (31/72) and 56.9% (41/72). 32 out of 41 treatment failures (78.0%) appeared within the first 3 months. Among the causative pathogens, Pseudomonas aeruginosa-related PJI had the poorest outcome resulting in an infection-free survival of only 18.2% (2/11), whereas infections caused by Enterobacter cloacae had the highest success rate of 58.3% (7/12); (*p* = 0.12). Success rates were 65.0% (13/20) for two-stage revision, 38.1% (8/21) for multi-stage revision, 36.0% (9/25) for DAIR and 16.7% (1/6) for single-stage revision. In total, 6 amputations, 2 knee arthrodesis, and 8 resection arthroplasties were required for definitive infection eradication.

**Conclusion:**

Gram-negative PJI may follow a more aggressive course with earlier and higher failure rates than previously thought. DAIR may be an option in selected cases, while two-stage revision showed a trend towards a more favorable infection-free survival in comparison to single- and multi-stage revisions. The results suggest pathogen-specific differences may influence outcomes and support individualized treatment strategies, warranting confirmation in large, prospective, multicenter studies.

## Introduction

Despite the generally positive outcomes of total joint arthroplasty (TJA), the procedure’s increasing frequency in recent years has been accompanied by a significant rate of complications, resulting in implant revision rates as high as 20% [1–3].

One of the most severe complications following TJA is a periprosthetic joint infection (PJI).

Among the various pathogens responsible for PJI, gram-negative species account for 5% to 15% of all PJI cases [4, 5]. While eradication rates between 50 and 90% have been reported in PJI with more common causative pathogens such as Staphylococcus aureus or coagulase-negative staphylococci [6–8], PJI due to gram-negative bacteria are considered more severe and are associated with poorer outcomes. However, heterogeneous success rates between 27% and 79% have been reported [5, 9, 10]. Several factors may explain the higher failure rates in comparison to gram-positive PJI, one being the increased frequency of complex resistance patterns [11]. Another one might be an inadequate choice of surgical treatment options for different types of gram-negative PJI, especially as GN PJI is often associated with compromised host status. There are many different factors influencing treatment recommendations in PJI, one being the differentiation between acute and chronic infections. For acute infections with short symptom durations, implant retention with the exchange of all mobile components is broadly recommended as it is assumed that no stable biofilm formation has taken place up to this point [12]. While this is broadly accepted as the most important factor for either implant retention or removal, a proper identification of patients suitable for DAIR is inevitable as many more risk factors are suspected. Several risk assessment tools have been proposed [13, 14], but have not considered the causative pathogen. Recent studies have shown a correlation between poorer outcomes for PJI caused by Staphylococcus aureus and implant retention, indicating a correlation between the causative pathogen and treatment outcome [12]. As gram-negative PJI has been associated with lower success rates in general, we aimed to analyse if characteristics for improved outcomes can be found.

Therefore, the aim of this study was (1) to perform an outcome analysis for gram-negative PJI and (2) to identify the most suitable surgical treatment strategy in eradicating gram-negative PJI.

## Materials and methods

Ninety-seven cases of PJI in total hip and knee arthroplasty (THA & TKA) caused by gram-negative pathogens treated at an academic tertiary referral center between 2010 and 2022 were retrospectively identified. Inclusion criterium was a minimum follow-up of 12 months. Twenty-five patients were lost to follow-up and were therefore excluded from further analysis.

PJI was defined following the EBJIS-criteria [15]. All data including regular patient demographics and detailed recording of the infection were drawn retrospectively from a comprehensive database for all PJI treated at our institution.

Severity of PJI was evaluated according to the JS-BACH-classification and divided into uncomplicated, complex or limited options cases [16].

According to the Tsukayama classification [17], the cases were divided into early acute (< 30 days after index arthroplasty), late (> 30 days after index arthroplasty, chronic symptoms), acute haematogenous PJI (> 30 days after index arthroplasty, acute symptoms) or presumably aseptic (positive intraoperative cultures).

In case of polymicrobial gram-negative PJI, one primary pathogen showing the highest bacterial load was defined for further analysis.

The gram-negative pathogens were further divided regarding potential drug-resistances. 3-MRGN pathogens were defined as showing resistances to 3 out of the 4 most important antibiotic classes for GN treatment (acylaminopenicillins; 3rd or 4th gen cephalosporins; fluoroquinolones (ciprofloxacin); carbapenems). 4-MRGN pathogens showed corresponding resistances to all 4 classes.

## Treatment algorithms

Prior to revision of the implant, all cases and respective treatment modalities were discussed with an interdisciplinary team as well as the patient. Team members were the responsible orthopaedic surgeon, a microbiologist, and a pharmacist.

Surgical treatment included DAIR, single-, two- or multi-stage-revision depending on the general health status of the patient and type of infection. In accordance with our institution’s PJI algorithm, indications for DAIR were short symptom durations of less than four weeks, indicating either an early acute PJI (index surgery < 30 days) or an acute haematogenous PJI (index surgery > 30 days). DAIR was chosen in six cases of chronic PJI when the patient was too unwell to undergo implant removal or when the size of the implant (e.g. a total femoral replacement) made staged exchange nearly impossible. In those salvage situations where infection eradication was not the primary goal, long-term suppressive antibiotics were prescribed, and establishing a stable sinus tract was discussed in detail and eventually performed.

On the other hand, implant removal was the preferred strategy when a chronic infection was present, and the patient was fit for surgery. A two-stage revision was the preferred approach for implant exchange, while a single-stage approach was reserved for cases where multiple surgeries would be unmanageable for the patient. A multi-stage approach was chosen in cases involving multi-drug resistant pathogens, fungal infections, or persistent signs of infection at the time of planned reimplantation (e.g. elevated or increasing CRP levels, or wound drainage). Two-stage revision involved a 6-week interval with an antibiotic-loaded polymethylmethacrylate spacer (Gentamycin, Vancomycin and/ or Meropenem) and systemic antibiotics. DAIR procedures explicitly included the replacement of all mobile components.

Following our treatment algorithm, antibiotic therapy was continued for a duration of 12 weeks. If infection eradication was not the primary goal or did not seem achievable, long term suppressive antibiotic therapy (SAT) for over one year was prescribed.

Intraoperatively, five standardised culture-biopsies, sonication of the implant and one histopathological biopsy were acquired.

### Outcome measures

Treatment was considered successful according to the Delphi-based International Multidisciplinary Consensus consisting of three features: (1) healed wound without fistula, drainage or pain, indicating infection eradication and no infection recurrence caused by the same organism strain, (2) no subsequent surgical intervention for infection after reimplantation surgery (PJI-revision-free survival) and (3) no occurrence of PJI-related mortality [18].

Second, necessary salvage procedures (amputation, resection arthroplasty or establishment of a stable fistula) after definitive PJI treatment were assessed.

## Ethics and statistical analysis

The study was approved by the local institution’s Ethics Committee (reference no. 714/20 S) and was conducted in accordance with the Helsinki Declaration.

Written patients’ consent was obtained in advance as part of the database. Follow-up took place through out-patient clinic visits.

Normally distributed variables are given as mean and standard deviation (SD), non-normally distributed variables as median and interquartile range (IQR). The Shapiro-Wilk–Test was used to assess whether the variables followed a normal distribution.

For non-normally distributed variables, Mann-Whitney-U test and t-test were performed for all continuous variables. Pearson’s Chi-Squared-test was performed for comparison of categorial variables. Values of α < 0.05 were considered to indicate statistical significance. Survival analysis was done using Kaplan Meier survival statistics. Log-rank-test and cox-regression were used for survival comparisons. Statistical analysis was carried out using IBM SPSS Statistics for Windows, version 31.0 (Armonk, New York: IBM Corporation).

## Results

### Demographics, microbiology and anti-infectious treatment

Seventy-two cases met the final inclusion criteria. Median (IQR) follow-up was 18.0 (46.0) months. Detailed patients’ demographics are demonstrated in Table 1.

Regarding the microbial spectrum, the detected pathogens were Escherichia coli (23/72), Klebsiella species (13/72), Enterobacter cloacae (12/72), Pseudomonas species (11/72), Proteus mirabilis (5/72) and several others, each only found in 1 or 2 cases (8/72). Of the isolates, 11.1% (8/72) were classified as 3-MRGN and 2.8% (2/72) as 4-MRGN. Another three had a single resistance to ciprofloxacin (4.2%). Polymicrobial infections associated with two or more pathogens were present in 58.3% of cases (42/72).

Vancomycin (blood-level controlled) and Meropenem (3x/d 2 g) were the most frequently used iv-antibiotics (35.7% and 34.3%), followed by Ampicillin/ Sulbactam (3x/d 3 g) (24.3%) and Piperacillin/ Tazobactam (3x/d 4.5 g) (15.7%). Iv-antibiotics were continued for a mean (SD) of 20.3 (10.5) days. For oral administration, Ciprofloxacin (2x/d 750 mg) was chosen in 76.0% of cases, followed by Rifampicin (2x/d 450 mg) (20.0%), Cotrimoxazole (3x/d 960 mg) (16.0%) and Levofloxacin (2x/d 500 mg) (12.0%). Oral antibiotics were continued for a mean (SD) total duration of 18.4 (4.7) weeks including SAT. SAT for a period of more than one year was prescribed in 13 cases (18.1%).

**Table 1:** Patients’ demographics and data on periprosthetic infections (IQR = interquartile range; JS-BACH = joint-specific, bone-involvement, anti-microbial options, coverage of the soft tissues, host status classification; PJI = periprosthetic joint infection)

Age in years (median; IQR)			72.0 (59–85)
**Follow-up in months (median; IQR)**			18.0 (46.0)
**Sex (n; %)**			
		Male	31/72 (43.1)
		Female	41/72 (56.9)
**Location (n; %)**			
		Knee	14/72 (19.4)
		Hip	58/72 (80.6)
**BMI in kg/m² (median; IQR)**			28.9 (22–36)
**JS-BACH-classification (n; %)**		Uncomplicated	17/72 (23.6)
		Complex	34/72 (47.2)
		Limited options	21/72 (29.2)
**Type of infection I (Tsukayama et al.) (n; %)**			
		Early acute	18/72 (25.0)
		Acute haematogenous	8/72 (11.1)
		Late chronic	46/72 (63.9)
**Implant (n; %)**		Primary TKA/ THA	33/72 (45.8)
		Revision TKA/ THA	29/72 (40.3)
		Megaprosthesis	10/72 (13.9)
**Prior PJI in history? (n; %)**			29/72 (40.3)
**Preoperative sinus tract (n; %)**			16/72 (22.2)
**Polymicrobial PJI (n; %)**			42/72 (58.3)
**Gram-negative pathogen (n; %)**			
	Escherichia coli		23/72 (31.9)
	Enterobacter cloacae		12/72 (16.7)
	Klebsiella spp.		13/72 (18.1)
	Proteus mirabilis		5/72 (6.9)
	Pseudomonas spp.		11/72 (15.3)
	Citrobacter spp.		2/72 (2.8)
	Brucella melitensis		1/72 (1.4)
	Acinetobacter baumanii		2/72 (2.8)
	Neisseria meningitidis		1/72 (1.4)
	Morganella morganii		2/72 (2.8)

### Overall outcome

The overall infection-free survival was 43.1% (31/72) after a mean of 27.0 months. 32 out of 41 treatment failures (78.0%) appeared within the first 3 months.

The PJI-revision-free survival was 56.9% (41/72). Of all revisions for recurring PJI, 80.6% (25/31) were performed within the first 3 months after the initial PJI.

The infection-free survival for late, chronic PJI was 45.6% (21/46), 38.9% (7/18) for early acute and 37.5% (3/8) for acute haematogenous PJI (*p* = 0.84).

Twelve patients died in the observed time-period (16.8%). In five cases (6.9%), the cause of death was directly associated to the ongoing PJI. There were 6 amputations of the affected extremity (8.3%), 2 knee arthrodesis (2.8%) and 8 resection arthroplasties of the hip joint (11.1%) for definitive infection control.

### Pathogen-specific outcome

Enterobacter cloacae PJI showed the highest infection-free survival of 58.3% (7/12), followed by Escherichia coli (43.5%; 10/23), Proteus mirabilis (40.0%; 2/5), Klebsiella spp. (30.8%; 4/13) and Pseudomonas spp. (18.2%; 2/11).

Poly- and monomicrobial infections did not differ in overall infection-free (42.9% vs. 43.3%; *p* = 0.97) or PJI-revision-free survival (46.7% vs. 64.3%; *p* = 0.14).

Regarding multi-drug-sensitive and -resistant pathogens, the infection-free survival rate was 45.8% (27/59) for sensitive pathogens, 33.3% (1/3) for a single resistance to ciprofloxacin (*p* = 0.68), 25.0% (2/8) for 3-MRGN (*p* = 0.08) and 50.0% (1/2) for 4-MRGN (*p* = 0.89).

**Table 2:** Analysis of treatment outcome regarding different prognostic factors using the Cox proportional hazards model. (A) univariate analysis for potential risk factors. (B) multivariate analysis adjusted for ASA grade, JS-BACH group, joint type, infection type and polymicrobial status. (GN = gram-negative; Ref. = reference; HR = hazard ratio)

		Infection-free success rate (%)	A) Univariate analysis			B) Multivariate analysis		
			HR	95%-CI	*p*-value	HR	95%-CI	*p*-value
**Type of surgical therapy**	**Two-stage**	65.0 (13/20)	Ref.	Ref.	Ref.	Ref.	Ref.	Ref.
	**Single-stage**	16.7 (1/6)	2.7	0.9–8.6	0.09	1.6	0.3–8.2	0.59
	**Multi-stage**	38.1 (8/21)	1.9	0.7–4.8	0.17	1.1	0.3–3.4	0.91
	**DAIR**	36.0 (9/25)	2.1	0.8–5.1	0.10	1.4	0.4–4.6	0.63
**Resistance pattern**	**Sensitive pathogens**	45.8 (27/59)	Ref.	Ref.	Ref.	Ref.	Ref.	Ref.
	**Multi-drug resistant GN pathogen**	30.8 (4/13)	1.6	0.7–3.3	0.21	1.8	0.8–4.1	0.17
**Type of GN pathogen**	**E. cloacae**	58.3 (7/12)	Ref.	Ref.	Ref.	Ref.	Ref.	Ref.
	**E. coli**	43.5 (10/23)	1.4	0.5–3.9	0.52	1.4	0.5–4.3	0.50
	**Proteus**	40.0 (2/5)	1.5	0.3–6.4	0.56	1.6	0.3-9.0	0.62
	**Klebsiella**	30.8 (4/13)	1.9	0.6–5.6	0.27	2.1	0.6-7.0	0.2
	**Pseudomonas**	18.2 (2/11)	2.4	0.8–7.2	0.12	3.0	0.8–11.5	0.10

### Therapeutical options for gram-negative PJI

**Fig. 1:**
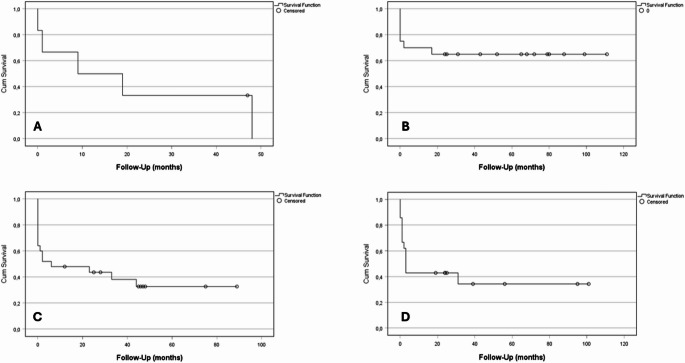
Kaplan-Meier analysis for infection-free survival regarding the surgical strategy: (A) single-stage revision (*n* = 6), (B) two-stage revision (*n* = 20), (C) DAIR (*n* = 25) and (D) multi-stage revision (*n* = 21). HR (95%-CI) with two-stage revision as reference was 2.7 (0.8–8.6) for single-stage revision, 2.1 (0.8–5.1) for DAIR and 1.9 (0.7–4.8) for multi-stage revision

Two-stage revision provided the highest overall infection-free survival of 65.0% (13/20). In comparison, multi-stage revision resulted in a success rate of 38.1% (8/21; *p* = 0.13), followed by DAIR (36.0%; 9/25; *p* = 0.06) and single-stage revision (16.7%; 1/6; *p* = 0.06); (Fig. 1; Table 2).

Out of the 46 cases of late, chronic PJI, 6 were treated with DAIR when major surgery was not bearable for the patient. In these cases, the infection- and revision-free survival was 16.7% (1/6) and 33.3% (2/6), respectively. For all implant removal strategies combined and in comparison, to DAIR, the infection- and revision-free survival was 50.0% (20/40; *p* = 0.05) and 67.5% (27/40; *p* = 0.06), respectively.

Considering acute infections, DAIR provided an infection- and revision-free survival of 42.1% (8/19) and 47.4% (9/19). When an implant removal was performed, success rates were 28.6% (2/7; *p* = 0.625) and 42.9% (3/7; 0.863), respectively.

### Further risk factors for treatment failure

The JS-BACH-classification was associated with worse infection-free survival for the limited options group (14.3%; 3/21) in comparison to complex (50.0%; 17/34; *p* = 0.007) and uncomplicated cases (64.7%; 11/17; *p* = 0.001).

An ASA-Score of three or higher was also associated with significantly lower success-rates (32.5% vs. 57.1%; *p* = 0.043).

Previous PJI in history, polymicrobial infection, the type of infection (Tsukayama and acute/chronic), a preoperative fistula, patients’ sex, location (hip/ knee) and obesity did not show any significant influence on the outcome.

## Discussion

### Outcome of Gram-negative PJI

The most important finding of this study is that PJI caused by gram-negative pathogens shows high and in particular early failure rates. With an overall success rate of just 43%, a revision-free survival rate of 57% and most failures occurring within the first three months after reimplantation or DAIR, these findings underscore the aggressive nature and poor prognosis of gram-negative PJI.

These results are further emphasized by the high rate of salvage procedures required in our cohort. Nearly every fourth patient ultimately underwent amputation, arthrodesis, or resection arthroplasty—highlighting not only the clinical complexity but also the severe limitations in therapeutic options for this patient group.

### Treatment strategies

While most authors agree on when to retain or remove implants in general and a recently published recommendation by the European Bone and Joint Infection Society (EBJIS) on when to perform a curative DAIR has been published [12], indications in specific situations and especially considering specific causative pathogens, remain unclear.

Reported success rates for gram-negative PJI vary widely (27–94%) [5, 19]. On average, a recent systematic review by Gonzalez et al. including 593 cases of GN PJI has found success rates of 66% for early acute, 39% for acute hematogenous and 75% for chronic infections with no significant differences between DAIR and implant removal success rates for either acute or chronic PJI in the overall cohort [20].

In contrast, our cohort showed better outcomes for chronic PJI when an implant removal was performed in comparison to implant retention – which is in line with recent experiences in general PJI treatment [12]. Two-stage revision yielded the highest infection-free survival of 65%, while DAIR and multi-stage revision had sobering success rates of 36% and 38%. One-stage revision was reserved for selected cases and showed poor results, most likely due to patients’ frailty as this approach was only chosen when patients were not able to undergo several extensive surgeries.

Hsieh et al. reported similarly poor outcomes of just 27% following GN PJI treated with debridement alone, but noted significantly improved results for cases treated with two-stage revision (87%) [5]. In contrast, the highest success rate of 94% had been reported by Aboltins et al. in a small cohort study of 17 cases of early acute PJI treated with DAIR within 7 symptomatic days [19]. Explanations for the widely ranging results might be the different indications for DAIR and the exact surgical procedure with or without exchange of all mobile parts. While Aboltins et al. had a highly selective cohort ideally suited for DAIR with the exchange of all mobile components (less than 7 days symptomatic, early postoperative PJI), Hsieh et al.’s and also our own low success rate might originate from a less selective cohort with longer symptom durations. In addition, Hsieh et al. did not perform an exchange of the mobile components in all cases which is a known risk factor for failed DAIR [12]. While our DAIR procedures consistently involved mobile-part exchange, the decision to retain the implant was often influenced by insufficient bone stock for reimplantation and severe comorbidities. Shah et al. have reported similar findings in a cohort of PJI associated with Pseudomonas spp [21]. They found a 26% success rate for implant retention after a two-year follow-up in comparison to 83% for two-stage revision, but they explicitly mentioned the high rate of chronic infections (> 2 weeks of symptoms) included as an explanation for the high failure rate.

Even when considering DAIR as a salvage procedure for patients who were not able to cope with an implant exchange, two thirds had to undergo at least one further revision for recurring PJI.

### Timing of failure

It is worth noting that besides the generally high failure rate in our cohort, there was a strikingly high rate of early failures and especially early surgical revisions for recurring PJI. Grossi et al. have shown similar results where 14 out of 16 failures appeared after a median of 21 days following definitive surgery and still being under antibiotic therapy [22]. Kalbian et al. reported early reoperations in polymicrobial PJI involving gram-negative pathogens, but not for mono-microbial gram-negative PJI [23].

Despite these results, we did not identify specific predictors for early failure, suggesting a generalized risk in GN PJI that warrants close postoperative monitoring of patients to detect early failure.

### Limitations

Several limitations of this study should be acknowledged. First, the study’s retrospective design inherently carries limitations, including potential biases and data collection challenges.

We willingly analysed all surgical treatment procedures and did not focus solely on DAIR as treatment decisions were made by the respective surgeon on a case-by-case basis. Consequently, a selection bias favouring DAIR as the more preserving and less invasive therapy option cannot be excluded. Second, the small cohort size may limit the generalizability of the findings to a broader patient population, especially considering the small subsets of cases. The study cohort was intentionally inclusive but consequently highly heterogeneous, comprising hip and knee PJIs, primary and revision implants, megaprostheses, mono- and polymicrobial infections, and various surgical strategies. This lack of homogeneity limits direct comparability between subgroups and results in substantial confounding by indication, particularly regarding surgical strategy selection, which should be considered when interpreting comparative outcome analyses. The high number of cases lost to follow-up restricts the scope of the study, potentially introducing attrition bias and reducing statistical power. This undermines the validity and generalisability of the observed outcomes. Successful cases in particular may not have adhered to regular follow-up, which could lead to better-than-expected results.

Last, the relatively short follow-up duration restricts our ability to draw conclusions about long-term clinical outcomes. However, a relevant number of infections have already recurred within a short interval, and we therefore decided not to exclude further cases.

## Conclusion

In conclusion, our study highlights the potentially aggressive course of gram-negative PJI, with a trend toward high and early failure rates. However, these findings should be interpreted cautiously given the limited sample size, lack of adjustment, and several non-significant results. While DAIR may be considered in carefully selected GN-related PJI cases, and two-stage revision appeared to be associated with more favorable infection-free survival, these observations reflect trends rather than definitive evidence. Overall, our data suggest that pathogen-specific differences may influence outcomes and support the need for more individualized treatment strategies, but robust conclusions require validation. Future prospective, high-volume, multicenter studies are essential to confirm these trends, clarify pathogen-specific effects, and better elucidate the sources and mechanisms of gram-negative PJI.

## Data Availability

The dataset supporting the conclusions of this article has not been published anywhere but can be made available on request.
